# Enhancing immunomodulation in cyclophosphamide‐induced immunosuppressed mice through targeted modulation of butyrate‐producing gut microbiota via oral administration of astragalus polysaccharides

**DOI:** 10.1002/fsn3.4386

**Published:** 2024-08-05

**Authors:** XinQian Rong, QingLong Shu

**Affiliations:** ^1^ College of Traditional Chinese Medicine Jiangxi University of Chinese Medicine Nanchang China

**Keywords:** astragalus polysaccharide, butyrate, butyrate‐producing bacteria, gut microbiota, immunosuppression

## Abstract

Astragalus polysaccharide is one of the most extensively studied traditional Chinese medicinal polysaccharides because of its immunomodulatory activity and has attracted considerable attention. Existing evidence suggests that its potential immunomodulatory mechanism is related to the modulation of intestinal microbiota. However, current research methods on the gut microbiota mainly focus on 16S rRNA sequencing, providing limited evidence of specific changes in functional bacterial groups in the intestine. Butyrate is a class of short‐chain fatty acids among the microbial metabolites in the gut and is most closely associated with immunomodulatory activity. Thus, in this study, we extracted and purified a polysaccharide from astragalus composed of a main chain of →4)‐α‐D‐Glcp‐(1 → and →4,6)‐α‐D‐Glcp‐(1→, with side chains of →6)‐α‐D‐Glcp‐(1→ and aggregated arabinose, and investigated the changes in butyrate‐producing bacterial groups in mice during the immunomodulation process of astragalus polysaccharide, using two butyrate‐producing bacterial‐specific primers. The results showed that oral administration of astragalus polysaccharide significantly increased butyrate production in the mouse intestine, restoring the disrupted butyrate‐producing bacterial abundance and diversity caused by immunosuppression. In conclusion, our study provides the first evidence of the targeted modulation of the butyrate‐producing gut microbiota by astragalus polysaccharide, offering insights into its pharmacological activity.

## Graphical abstract

In this study, we extracted and purified a polysaccharide from Astragalus composed of a main chain of →4)‐α‐D‐Glcp‐(1→ and →4,6)‐α‐D‐Glcp‐(1→, with side chains of →6)‐α‐D‐Glcp‐(1→ and aggregated arabinose, and investigated the changes in butyrate‐producing bacterial groups in mice during the immunomodulation process of Astragalus polysaccharide, using two butyrate‐producing bacterial‐specific primers. The results showed that oral administration of Astragalus polysaccharide significantly increased butyrate production in the intestines of the mice, restoring the disrupted butyrate‐producing bacterial abundance and diversity caused by immunosuppression.

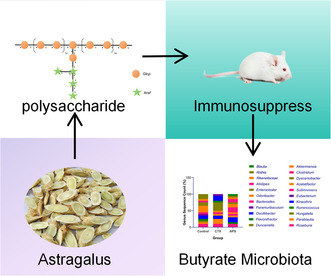

## 1 INTRODUCTION

Astragalus, derived from the roots of the perennial herbaceous plants Astragalus mongholicus and Astragalus membranaceus of the legume family, can be used as food or medicine (Balakrishnan et al., 2021). In traditional Chinese medicine, astragalus has been used for thousands of years as a classic tonic medicine and is widely employed in diseases related to immune deficiency (Fu et al., 2014). Modern pharmacological studies have shown that astragalus polysaccharides is one of the most important pharmacological components of astragalus, exerting stimulatory effects on immune responses in cancer patients (Zhang, Wu, et al., 2023) and immune‐suppressed animal models (Li et al., 2023). Several studies have suggested that the class of macromolecular polysaccharides represented by astragalus polysaccharides is poorly digested by the human body, likely due to a lack of genes encoding enzymes for certain complex carbohydrates. These indigestible polysaccharides enter the human colon, where they are degraded and utilized by resident gut microbes to produce active metabolites that maintain gut homeostasis (Zhao et al., 2023). These metabolites, notably short‐chain fatty acids, are a significant source of pharmacological activity against macromolecular polysaccharides.

Butyric acid is one of the most important short‐chain fatty acids in the human gut. Studies have suggested that a deficiency of butyric acid in the gut can increase the risk of inflammation (Tian et al., 2022), and that enhancing the production of butyric acid by gut microbes promotes activation of the human immune system (He et al., 2021). Butyric acid serves as an energy source for the intestinal barrier system and gut microbiota, and also acts as a bioactive substance in immune activation in the human body (Fu et al., 2019). Therefore, when exploring the relationship between immune‐related diseases and the gut microbiota, changes in the functional microbial community that produce butyric acid in the gut are pivotal factors to consider.

Currently, in studies related to polysaccharide‐microbiota‐metabolite axis correlations, the monitoring method for changes in the microbial community often involves 16S rRNA sequencing (Cai et al., 2024). However, owing to the inherent limitations of sequencing methods, 16S rRNA sequencing cannot comprehensively enrich and characterize changes in a specific functional microbial community (Xiao et al., 2022). Hence, finding a comprehensive enrichment method for genes of a specific functional microbial community in the gut microbiota is of paramount importance in gut microbiota research. For butyric acid‐producing functional microbial communities, two types of genes are directly related to their production: butyryl‐CoA: acetate CoA‐transferase (EC:2.8.3.8 2.8.3.9) and butyrate kinase (EC:2.7.2.7), using enrichment primers published in studies by Louis and Flint (2007) and Meng and Shu (2024), respectively.

Therefore, in this study, we extracted and purified a macromolecular astragalus polysaccharide (APS‐E1F1), investigated its oral therapeutic effects in cyclophosphamide‐induced immunosuppressed mice, and used two butyric acid‐producing functional gene primers to enrich the gut microbiota. We then characterized changes in the butyric acid‐producing functional microbial community in the gut during the treatment process. This study serves as a starting point to explore the gut microbiota mechanisms that represent changes in functional microbial communities during astragalus polysaccharide immune activation, and aims to provide a partial foundation for further pharmacological research on astragalus polysaccharides, offering insights into the polysaccharide‐microbiota‐metabolite axis.

## 2 MATERIALS AND METHODS

### 2.1 Chemicals and reagents

Astragalus was sourced from Jiangxi Jiangzhong Chinese Medicine Decoction Pieces Co., Ltd. (Yingtan, China). Monosaccharide standards and dextran were obtained from Sigma‐Aldrich (St. Louis, MO, USA). Cyclophosphamide (CTX) was purchased from Aladdin (Shanghai, China). The fructooligosaccharides were purchased from SolarBio (Beijing, China). Enzyme‐linked immunosorbent assay (ELISA) kits for immunoglobulins and cytokines, including IgG, IgM, interleukin 2 (IL‐2), interleukin 6 (IL‐6), and tumor necrosis factor α, were sourced from Ruixin Biological Technology Co., LTD (Quanzhou, China). The hematoxylin and eosin (H&E) staining kit was acquired from Beyotime Biotechnology (Shanghai, China). Other chemical reagents and solvents were of analytical grade and were purchased from Sino Pharm Co. (Shanghai, China).

### 2.2 Extraction and purification of polysaccharides from astragalus

**FIGURE 1:**
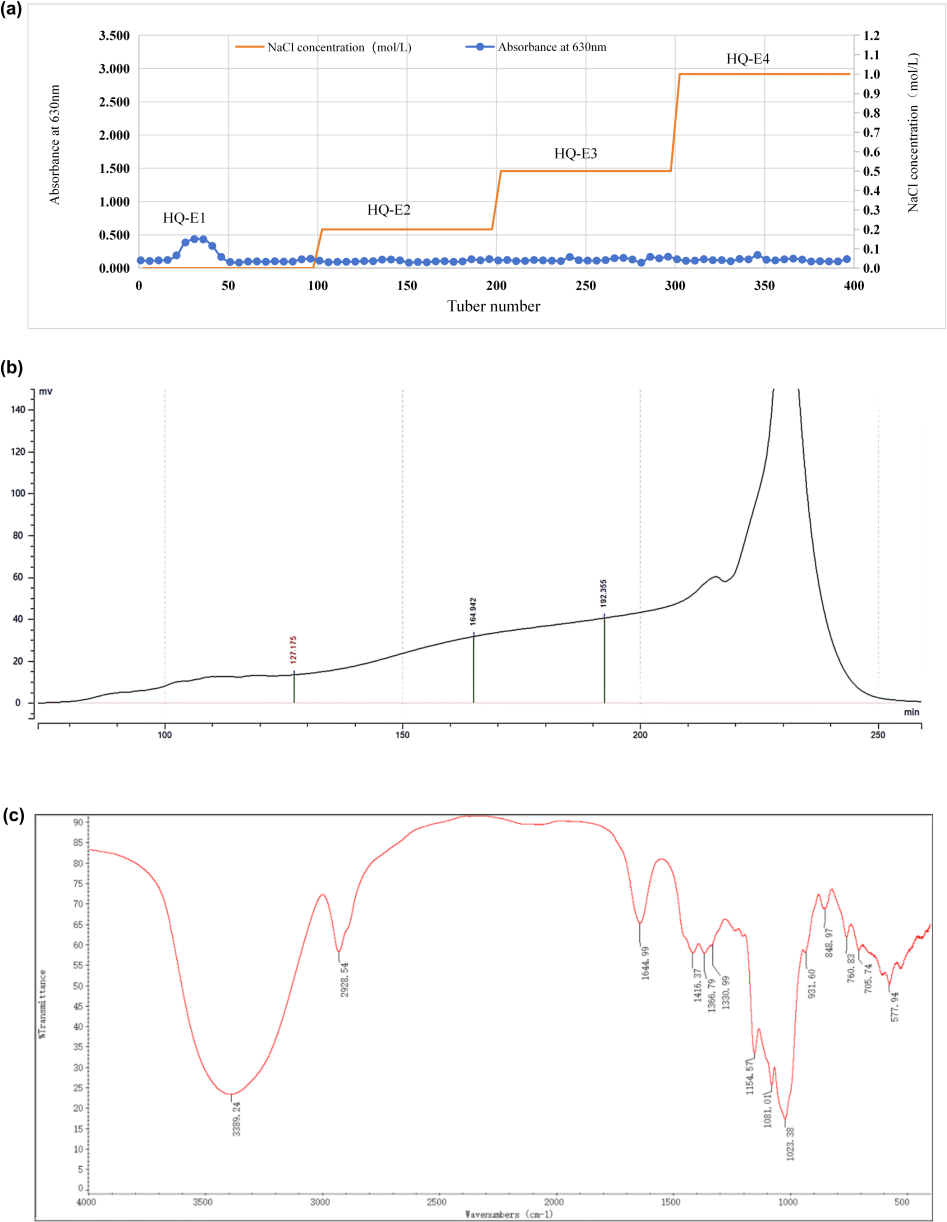
APS‐E1F1 elution and components. (a) Elution curve of APS by DEAE‐52 ion exchange column chromatography. (b) Elution curve of APS‐E1 by Sephacryl S‐400 HR column. (c) Fourier transform infrared spectrum (FT‐IR) measurement.

Polysaccharides from Astragalus were extracted via water extraction and alcohol precipitation. Astragalus was soaked in water (1:6, w/v) for 20 min, followed by a 30 min decoction. The resulting filtrate was collected, and the residue was subjected to decoction in water (1:4, w/v) for 20 min. Subsequently, the two filtrates were combined, concentrated, and treated with Sevage reagent to remove the proteins. Macroporous resin (AB‐8) was then used to remove the pigments. Membrane dialysis was employed to remove small molecules and purify the Astragalus polysaccharide. The polysaccharide sample was dissolved in an appropriate amount of dH2O, centrifuged at 8000 rpm for 10 min to remove precipitates, filtered through a 0.45 μm microporous membrane, and loaded onto a pre‐equilibrated ion exchange chromatography column (DEAE sepharose F) with an application volume of 30% of the column volume. After the entire sample had entered the column, elution was carried out sequentially with different salt concentrations (dH_2_O, 0.2 M NaCl, 0.5 M NaCl, 1.0 M NaCl) at two column volumes for each concentration, eluting at a flow rate of 15 mL/min. The fractions were collected using an automated fraction collector, with 100 tubes collected per elution gradient, each containing 10 mL. The polysaccharide content in the eluate was measured at 630 nm using the anthrone‐sulfuric acid method. The polysaccharide elution curve was obtained by plotting the number of tubes on the x‐axis and the absorbance on the y‐axis (Figure 1a). The HQE1 fractions were concentrated under vacuum, dialyzed with a 3.5 kDa cutoff membrane, and freeze‐dried under vacuum, resulting in various components. The primary components were selected for further purification. They were dissolved in an appropriate amount of dH2O, centrifuged at 8000 rpm for 10 min to remove precipitates, filtered through a 0.45 μm microporous membrane, and then loaded onto a pre‐equilibrated gel filtration chromatography column with an application volume of 1% of the column volume. After all the samples entered the column, elution was performed with ultrapure water at a flow rate of 1.8 mL/min. The eluate was collected using an automatic fraction collector with an elution of one column volume. The eluate from the region of the elution peak with high peak height and good symmetry was collected using an online differential detector (Figure 1b). Gel filtration column chromatography was repeated several times to enrich and purify the polysaccharides (APS‐E1F1) (Zhang, Li, et al., 2021).

### 2.3 Structure characterization of APS‐E1F1

#### 2.3.1 Determination of homogeneity and molecular weight

To evaluate homogeneity and molecular weight, polysaccharide samples were dissolved in a 0.1 M NaNO_3_ aqueous solution containing 0.02% NaNO_3_ at a concentration of 1 mg/mL. Subsequently, filtration through a 0.45 μm filter was performed. The filtered solution was then dissolved in a DMSO solution with 0.5% w/w lithium bromide (DMSO/LiBr) at the same concentration and underwent another filtration step using a 0.45 μm filter. The assessment of homogeneity and molecular weights of the fractions was carried out using SEC‐MALLS‐RI, with data analysis performed using ASTRA 6.1 software.

#### 2.3.2 Monosaccharide composition analysis

For monosaccharide composition analysis, approximately 5 mg of the sample was hydrolyzed with 2 M trifluoroacetic acid at 60°C for 30 min within a sealed tube. The resultant material was subsequently dried under nitrogen, washed with methanol, and then redissolved in deionized water after multiple washes. This solution was filtered through a 0.22 μm microporous filter for further analysis. The sample extracts were evaluated using high‐performance anion‐exchange chromatography (HPAEC) on a CarboPac PA‐20 column (3 × 150 mm; Dionex, Sunnyvale, CA, USA) equipped with a pulsed amperometric detector (Dionex ICS 5000+ system). Data processing for both standard and sample curves was conducted using Chromeleon software.

#### 2.3.3 Infrared spectroscopy analysis

Two milligram samples of Astragalus polysaccharides were mixed with 200 mg of potassium bromide powder in a mortar and ground evenly to form pellets for analysis. Infrared spectra were collected using a Fourier‐transform infrared spectrometer, scanning from 4000 to 400 cm^−1^, and the obtained spectra were documented.

#### 2.3.4 Methylation analysis

The polysaccharide samples underwent dissolution in DMSO, methylation in DMSO/NaOH with methyl iodide, and subsequent acquisition of permethylated products. Following this, the products were subjected to hydrolysis with 2 mol/L TFA at 60°C for 1 h, reduction using NaBD4, and acetylation with acetic anhydride for 2.5 h at 100°C. The resulting acetates were dissolved in chloroform and analyzed via gas chromatography–mass spectrometry (GC–MS) using an Agilent 6890A–5975C system equipped with an Agilent BPX70 chromatographic column (30 m × 0.25 mm × 0.25 μm, SGE, Australia). High purity helium served as the carrier gas with a split ratio of 10:1, and an injection volume of 1 μL. Mass spectrometry analysis commenced at 140°C for 2.0 min, followed by a temperature ramp of 3°C/min until reaching 230°C, where it was held for 3 min. The SCAN mode was employed for mass spectrometry, covering a range (*m/z*) of 50–350 *m/z*.

#### 2.3.5 Nuclear magnetic resonance spectroscopy analysis

The samples were dissolved in 0.5 mL D_2_O at a final concentration of 40 mg/mL. Subsequently, 1D‐NMR and 2D‐NMR spectra (1H‐NMR, 13C‐NMR, DEPT‐135, 1H‐1H COSY, HSQC, HMBC, NOESY) were recorded at 25°C utilizing a Bruker AVANCE NEO 600 M spectrometer operating at 600 MHz.

### 2.4 Animal experiment

#### 2.4.1 Modeling methods and treatments

BALB/c mice of male‐specific pathogen‐free grade, weighing 20 ± 2 g and aged 6–8 weeks, were sourced from Hunan Slac Jingda Laboratory Animal Co., Ltd. (SCXK(Xiang)2021‐0002; Hunan, China). All animal experiments were approved by the Experimental Animal Ethics Committee of Jiangxi University of Traditional Chinese Medicine (Ethics Committee No. JZLLSC20240306004) and conducted in accordance with established protocols and animal welfare guidelines. The research facility followed the principles set out in the Guide for the Care and Use of Laboratory Animals. After a 10‐day acclimatization period, the mice were divided into four groups (*n* = 8 each): control, model (CTX), and Astragalus polysaccharides treatment group (APS). For three consecutive days, mice in the CTX and APS groups were given intraperitoneal injections of Cyclophosphamide (CTX) at 100 mg/kg, while those in the control group received a saline equivalent. After 3 days, CTX group mice underwent natural recovery, while APS group mice received oral gavage of Astragalus polysaccharides at 200 mg/kg for seven days. On the eighth day, the mice were euthanized. The immunodeficiency model was established following a method adapted from Yu et al. (2021), and the dosage of polysaccharides was based on Li et al. (2023), with preliminary experiments determining the optimal dosages of CTX and polysaccharides for oral administration.

#### 2.4.2 Measurement of immunological indicators

Plasma samples were analyzed for immunoglobulin (IgM and IgG) and cytokine (IL‐2 and IL‐6) concentrations using commercial ELISA kits as per the manufacturer's instructions. The spleen and thymus were collected to calculate the splenic and thymic indices.

#### 2.4.3 Gut morphology analysis

Colon tissues were fixed in 4% paraformaldehyde, embedded in paraffin, and sectioned into 5 μm slides. Following deparaffinization and rehydration, slides were stained using an H&E staining kit (Beyotime) and examined for morphological changes with a Leica DFC310 FX digital camera connected to a Leica DMI4000B light microscope (Leica, Wetzlar, Germany).

#### 2.4.4 Measurement of butyric acid levels in feces

After the intervention, fecal samples were collected from each group in sterile EP tubes and resuspended by adding 50 μL of 20% phosphoric acid with 500 μM of 4‐methylpentanoic acid as an internal standard. Following centrifugation at 14,000×*g* for 20 min, the resulting supernatant was transferred to an injection vial for analysis using GC–MS with an injection volume of 1 μL and a 10:1 split ratio. Separation was carried out on an Agilent DB‐FFAP column (30 m × 250 μm × 0.25 μm) with an initial temperature of 90°C, ramping up at 10°C/min–160°C, further increasing at 40°C/min–240°C, and maintained for 5 min. Helium served as the carrier gas at a flow rate of 1.0 mL/min. Periodic inclusion of quality control samples was done for system assessment. Mass spectrometry analysis was performed using an Agilent 5977 B MSD mass spectrometer under specific conditions: inlet temperature at 250°C, ion source temperature at 230°C, transfer line temperature at 250°C, and quadrupole temperature at 150°C. An electron bombardment ionization source with an electron energy of 70 eV was utilized. Levels of butyric acid in fecal samples were quantified using the SCAN/SIM mode.

#### 2.4.5 Enrichment of butyric acid‐producing microbiota in the intestine

DNA samples were extracted following the procedure outlined in section 2.4.4. Primers BCo ATscr F (5′‐GCIGAICATTTCACITGGA AYWSITGGCAYATG‐3′) and BCo ATscr R (5′‐CCTGCCTTTGCAATRTCIACRAANGC‐3′) (Louis & Flint, 2007) were employed for PCR amplification of the butyryl‐CoA, acetate CoA‐transferase gene on the extracted fecal DNA. Additionally, the butyrate kinase primers BUK‐73‐FB (5′‐AANCCNGGHTCNACNTCNAC‐3′) and BUK‐638‐RB (5′‐CCNCCNCCNADRTG‐3′) were utilized (Meng & Shu, 2024). The PCR reactions consisted of 0.25 μL Ex Taq HS DNA polymerase (Takara, Beijing, China), 5 μL 109 Ex Taq Buffer, 4 μL dNTP, 1 μL of each primer, and 2 μL template DNA per 50 μL reaction. The PCR process was conducted using the EASYCYCLER 96 PCR instrument (Analytik Jena GmbH, Jena, Germany) with the following cycling conditions: initial denaturation at 95°C for 3 minutes, followed by 40 cycles of denaturation at 95°C for 30 s, annealing at 53°C for 30 s, and extension at 72°C for 30 s. A final extension step was performed at 72°C for 10 m, followed by storage at 4°C. PCR products were visualized by electrophoresis on a 1% agarose gel and subsequently purified. Post construction of the clone library, PCR products were inserted into the pEasy‐T1 vector (TransGen Biotech Co., Ltd., Beijing, China) and transformed into competent E. coli Trans1‐T1 cells. Fifty clones from each sample were randomly chosen for sequencing on an ABI3707 sequencer (ABI, Leuven, Belgium) using M13F and M13R vector primers based on sequence similarities.

### 2.5 Statistical analysis

The experimental results were expressed as mean ± SD. Equal variance was confirmed using SPSS Statistics v25 (SPSS Inc., Chicago, IL, USA). Group variances were compared using one‐way ANOVA with Bonferroni post‐hoc testing, with statistical significance set at *p* < .05. Graphical representation was created using GraphPad Prism software (version 8.0).

## 3 RESULTS

### 3.1 Molecular weight of APS‐E1F1

The number‐average molecular weight (Mn) reflects the arithmetic average molecular weight of a substance and indicates changes in the overall polysaccharide chain structure. The weight‐average molecular weight (Mw) emphasizes the effect of larger polysaccharide chains on the Mw distribution and polymer properties. For the astragalus polysaccharide APS‐E1F1, the Mn and Mw were assessed as 5.481 and 7.575 kDa, respectively.

### 3.2 Monosaccharide composition of APS‐E1F1

The monosaccharide composition of APS‐E1F1 was analyzed using HPAEC. Figure 1a shows the standard and sample curves. APS‐E1F1 predominantly comprised Man, Glc, Gal, and Ara in a ratio of 0.367:83.011:1.221:15.401.

### 3.3 Infrared spectroscopy analysis of APS‐E1F1

The infrared spectroscopy analysis revealed (Figure 1c) characteristic polysaccharide absorption peaks in the polysaccharide samples: a strong and broad absorption peak at 3389.24 cm^−1^ corresponding to the intermolecular or intramolecular hydrogen bond O–H stretching vibration; a medium intensity peak near 2928.54 cm^−1^ representing the C–H stretching vibrations of methyl (‐CH_3_) and methylene (‐CH_2_) groups; an absorption peak at 1644.99 cm^−1^ indicating the C=O stretching vibration of carbonyl groups or the presence of crystalline water; peaks in the range of 1450–1200 cm^−1^ at 1416.37 cm^−1^, 1366.79 cm^−1^, 1330.99 cm^−1^ representing bending vibrations of C–H bonds, which along with C–H stretching vibrations constitute characteristic sugar ring absorptions; a peak at 1154.57 cm^−1^ corresponding to the stretching vibration of pyranose C–O–C bonds; two strong absorption peaks between 1150 and 1010 cm^−1^ at 1080.01 cm^−1^ and 1023.38 cm^−1^, further confirming the presence of pyranose glycosides, with these absorptions corresponding to the bending vibrations of C–O bonds in C–O–H or C–O–C structures; a peak at 931.60 cm^−1^ representing vibrations of the sugar molecule; a peak at 848.97 cm^−1^ indicating the C–H bending vibrations of the alpha‐anomeric form of pyranose glycosides; and a peak at 760.83 cm^−1^ showing the symmetric ring stretching vibration of pyranose rings.

### 3.4 Methylation analysis of APS‐E1F1

**FIGURE 2:**
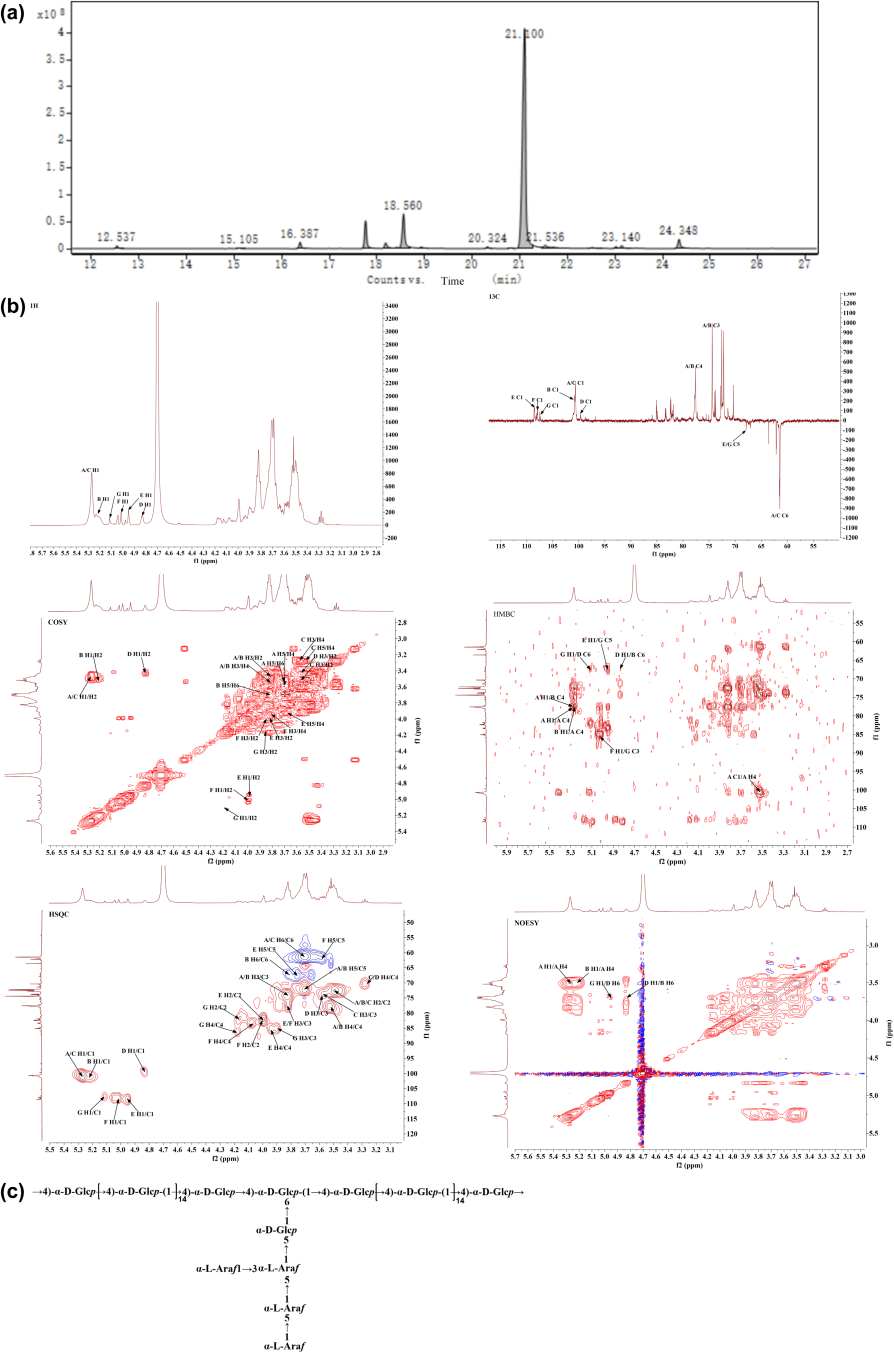
Structural analysis of APS‐E1F1. (a) Methylation analysis of APS‐E1F1. (b) APS‐E1F1 NMR maps. (c) Structural formula of APS‐E1F1.

Methylation analysis is essential for determining the type and proportion of glycosidic linkages. Based on the relative retention times of each chromatographic peak and mass spectrum (Figure 2a), comparison with literature data and the ion fragments mass spectrum from the Complex Carbohydrate Research Center database at the University of Georgia (https://glygen.ccrc.uga.edu/ccrc/specdb/ms/pmaa/pframe.html), the polysaccharide residue types corresponding to each chromatographic peak were analyzed and sequentially identified as t‐Ara(f), 5‐Ara(f), T‐Glcp, 3,5‐Ara(f), 4‐Gal(p), 4‐Glc(p), 6‐Glc(p), 3,4‐Glc(p), 4,6‐Glc(p). The most abundant glycosidic linkage type was 4‐Glc(p) with a content ratio of 84.167%. For more detailed information, please refer to Table 1.

**TABLE 1:** Results of polysaccharide residue data analysis.

Glycosidic linkage	PMAA	RT(min)	MW	Relative molar ratio (%)
t‐Ara(f)	1,4‐di‐O‐acetyl‐2,3,5‐tri‐O‐methyl arabinitol	12.537	279	0.882
5‐Ara(f)	1,4,5‐tri‐O‐acetyl‐2,3‐di‐O‐methyl arabinitol	16.387	307	1.952
T‐Glcp	1,5‐di‐O‐acetyl‐2,3,4,6‐tetra‐O‐methyl glucitol	17.76	323	7.748
3,5‐Ara(f)	1,3,4,5‐tetra‐O‐acetyl‐2‐O‐methyl arabinitol	18.932	335	0.304
4‐Gal(p)	1,4,5‐tri‐O‐acetyl‐2,3,6‐tri‐O‐methyl galactitol	20.806	351	0.210
4‐Glc(p)	1,4,5‐tri‐O‐acetyl‐2,3,6‐tri‐O‐methyl glucitol	21.1	351	84.167
6‐Glc(p)	1,5,6‐tri‐O‐acetyl‐2,3,4‐tri‐O‐methyl glucitol	21.536	351	1.511
3,4‐Glc(p)	1,3,4,5‐tetra‐O‐acetyl‐2,6‐di‐O‐methyl glucitol	23.025	379	0.456
4,6‐Glc(p)	1,4,5,6‐tetra‐O‐acetyl‐2,3‐di‐O‐methyl glucitol	24.348	379	2.770

### 3.5 NMR analysis of APS‐E1F1

To further elucidate the structural features of the polysaccharide samples, one‐dimensional nuclear magnetic resonance (1H‐NMR), DEPT‐135, and two‐dimensional nuclear magnetic resonance (1H‐1H COSY, HSQC, HMBC, and NOESY) analyses were conducted. The corresponding spectra are shown in Figure 2b, providing comprehensive proton and carbon chemical shift information for the major sugar residues (Table 2) and inferring the connectivity sequence between sugar residues.

**TABLE 2:** Attribution of 1H and 13C chemical shifts to individual sugar residues in APS‐E1N1.

Polysaccharide residues		Chemical shifts(ppm)							
			1	2	3	4	5	6a	6b
A	→4)‐α‐D‐Glcp‐(1→	H	5.27	3.49	3.82	3.52	3.7	3.7	3.58
		C	100.64	72.55	74.44	77.81	72.16	61.44	
B	→4,6)‐α‐D‐Glcp‐(1→	H	5.21	3.5	3.82	3.52	3.7	3.81	3.7
		C	101.03	72.62	74.44	77.83	72.16	67.13	
C	α‐D‐Glcp‐(1→	H	5.26	3.49	3.56	3.31	3.54	3.7	3.63
		C	100.62	72.55	73.33	70.21	71.47	61.44	
D	→6)‐α‐D‐Glcp‐(1→	H	4.84	3.45	3.57	3.31	3.7	3.71	3.8
		C	99.32	72.71	73.61	70.41	72.11	67.14	
E	→5)‐α‐L‐Araf‐(1→	H	4.95	3.98	3.82	3.94	3.66	3.75	
		C	108.64	81.94	77.74	85.23	67.54		
F	α‐L‐Araf‐(1→	H	5.02	3.99	3.87	4.06	3.58	3.69	
		C	108.12	82.45	77.76	85.23	62.09		
G	→3,5)‐α‐L‐Araf‐(1→	H	5.11	4.17	3.89	4.18	3.66	3.75	
		C	107.35	82.23	85.27	85.87	67.56		

In the 1H‐NMR spectrum of the polysaccharide sample, a large number of proton resonances were concentrated in the *δ* 3.0–5.5 ppm region, showing severe signal overlap. Multiple anomeric proton signals were observed in the *δ* 4.5–5.5 ppm region, indicating the presence of various sugar residues. The DEPT‐135 spectrum showed fewer signals, with multiple anomeric carbons in the *δ* 90–110 ppm region, suggesting the presence of arabinose residues. The strongest signal at *δ* 100.64 ppm was assigned to a glucose residue. In the *δ* 60–64 ppm region, there were apparent anti‐phase signals indicating the presence of –CH_2_ groups, and in the *δ* 65–70 ppm range, anti‐phase signals suggested substitution at the –CH_2_ position.

By analyzing the long‐range correlation signals in the HMBC spectrum between the protons on one residue and the carbons on various sugar residues or between the carbons on one residue and the protons on various sugar residues, one can further infer the connectivity sequence between the different sugar residues. In the HMBC correlation spectrum of the sample, the following coupling signals can be found: the coupling signal between H‐1 (*δ* 5.27 ppm) of residue A and C‐4 (*δ* 77.81 ppm) of residue A (A H‐1/A C‐4) indicates the presence of (a → 4)‐α‐D‐Glcp‐(1→) self‐connection, with the connection point at C‐4; the coupling signal between H‐1 (*δ* 5.27 ppm) of residue A and C‐4 (*δ* 77.83 ppm) of residue B (A H‐1/B C‐4) indicates the presence of (→4)‐α‐D‐Glcp‐(1→ and →4,6)‐α‐D‐Glcp‐(1→), with the connection point at C‐4; the coupling signal between H‐1 (*δ* 5.21 ppm) of residue B and C‐4 (*δ* 77.81 ppm) of residue A (B H‐1/A C‐4) indicates the presence of (→4,6)‐α‐D‐Glcp‐(1→ and →4)‐α‐D‐Glcp‐(1→), with the connection point at C‐4. In the NOESY correlation spectrum of the sample, the following coupling signals can be found: the coupling signal between H‐1 (*δ* 5.27 ppm) and H‐4 (*δ* 3.52 ppm) of residue A (A H‐1/A H‐4); the coupling signal between H‐1 (*δ* 5.21 ppm) of residue B and H‐4 (*δ* 3.52 ppm) of residue A (B H‐1/A H‐4). The coupling signal between H‐1 (*δ* 5.11 ppm) of residue G and C‐6 (*δ* 67.14 ppm) of residue D (G H‐1/D C‐6) indicates the presence of (a → 3,5)‐α‐L‐Araf‐(1→ and →6)‐α‐D‐Glcp‐(1→) connection, with the connection point at C‐6; the coupling signal between H‐1 (*δ* 4.95 ppm) of residue E and C‐5 (*δ* 67.54 ppm) of residue G (E H‐1/G C‐5) indicates the presence of (→5)‐α‐L‐Araf‐(1→ and →3,5)‐α‐L‐Araf‐(1→) connection, with the connection point at C‐5; the coupling signal between H‐1 (*δ* 4.84 ppm) of residue D and C‐6 (*δ* 67.13 ppm) of residue B (D H‐1/B C‐6) indicates the presence of (→6)‐α‐D‐Glcp‐(1→ and →4,6)‐α‐D‐Glcp‐(1→) connection, with the connection point at C‐6. In the NOESY spectrum, the coupling signal between H‐1 (*δ* 5.02 ppm) of residue F and C‐3 (*δ* 85.27 ppm) of residue G (F H‐1/G C‐3) indicates the presence of α‐L‐Araf‐(1→ and →3,5)‐α‐L‐Araf‐(1→) connection, with the connection point at C‐3; the coupling signal between H‐1 (*δ* 5.11 ppm) of residue G and H‐6 (*δ* 3.71 ppm) of residue D (G H‐1/D H‐6); the coupling signal between H‐1 (*δ* 4.84 ppm) of residue D and H‐6 (*δ* 3.81 ppm) of residue B (D H‐1/B H‐6).

Based on sugar composition analysis, methylation analysis, and the NMR data, the polysaccharide sample was tentatively identified as a polysaccharide with a main chain composed of (→4)‐α‐D‐Glcp‐(1→ and →4,6)‐α‐D‐Glcp‐(1→) and side chains composed of (→6)‐α‐D‐Glcp‐(1→) and polymerized arabinose. A possible repeating unit structure is shown in Figure 2c.

### 3.6 APS‐E1F1 exhibits immunomodulatory effects

**FIGURE 3:**
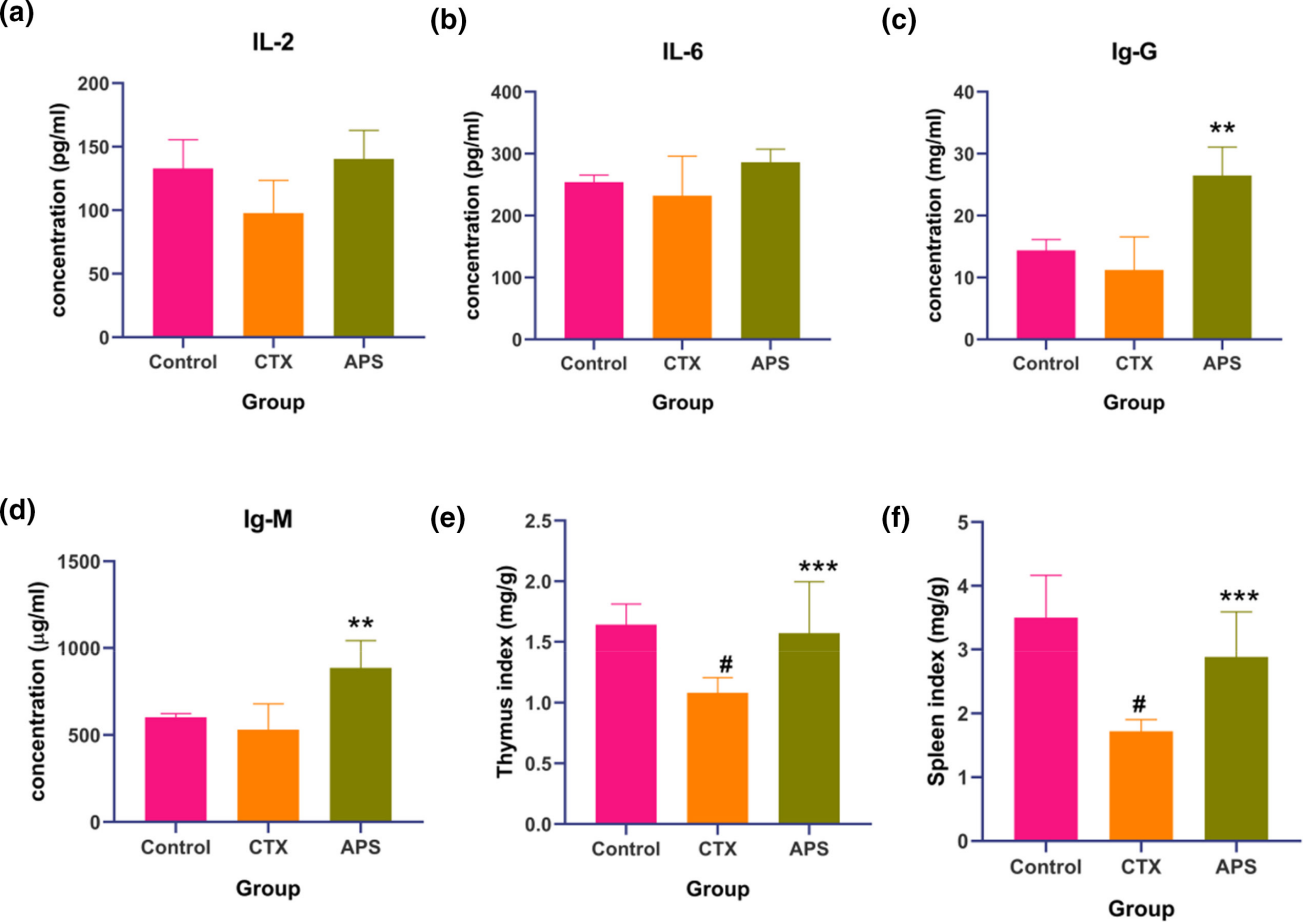
Plot illustrating the regulation of immune indicators in immunosuppressed mice by APS‐E1F1. (a, b) Immunoglobulin production in blood, including IgG and IgM. (c, d) Inflammatory cytokine secretion in blood, including IL‐2 and IL‐6. (e, f) thymus indexes and spleen indexes. Data were presented as mean ± SD (*n* = 8). #*p* < .05, Control group versus CTX group, **p < .01, ***p < .001, CTX group versus APS group.

Immunomodulation is an essential pharmacological activity of astragalus polysaccharides. In this study, we primarily assessed the serum levels of immunoglobulins (IgM and IgG) and cytokines (IL‐2 and IL‐6) (Figure 3a–d). The results indicated that APS had a varying degree of recovery effect on the decreased levels of immunoglobulins and cytokines induced by CTX, with a more pronounced recovery effect observed for immunoglobulins, showing significant differences (*p* < .05). Regarding organ indices, the CTX injection resulted in severe damage to the thymus (Figure 3e), and spleen (Figure 3f) in mice, which are challenging to recover naturally. Oral administration of APS partially reversed this decline in immune‐related organ indices, with statistically significant differences (*p* < .05).

### 3.7 APS‐E1F1 enhances intestinal barrier integrity as well as the quantitative level of butyric acid in the intestines

**FIGURE 4:**
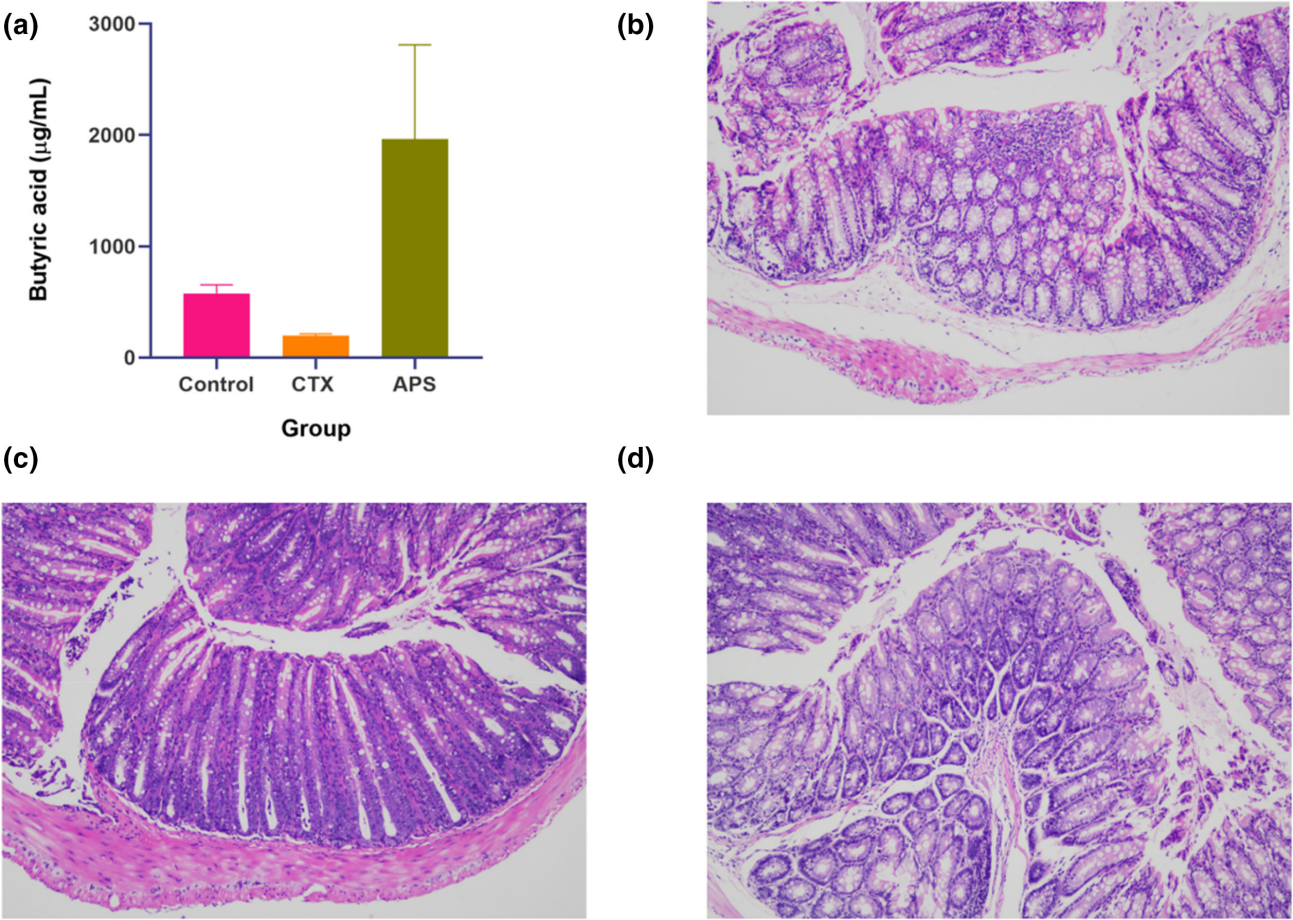
Quantification of butyric acid and intestinal barrier repair by APS‐E1F1. (a) Differences in butyric acid in different groups. (b–d) Observation of HE staining of mouse colon tissue sections. Magnification: 100×. Control group, CTX group, and APS group, in that order.

Butyric acid is a class of metabolites from the intestinal flora that is highly relevant to human immunity and is closely linked to intestinal barrier integrity. Therefore, we first determined the levels of butyric acid in the intestine (Figure 4a). The results showed that CTX injection affected the production of butyric acid in the intestines, leading to a decreasing trend in quantitative measurements. Oral administration of APS completely reversed this trend, resulting in a significant increase in the levels of butyric acid in the intestine, surpassing the normal levels found in control mice. We believe that a significant increase in butyric acid levels in the intestine improves the intestinal barrier. Histological staining of mouse colon tissues (Figure 4b–d) demonstrated that APS oral treatment fully repaired the intestinal barrier damage caused by CTX injection.

### 3.8 APS‐E1F1 modulates the structure of butyrate‐producing gut microbiota

**FIGURE 5:**
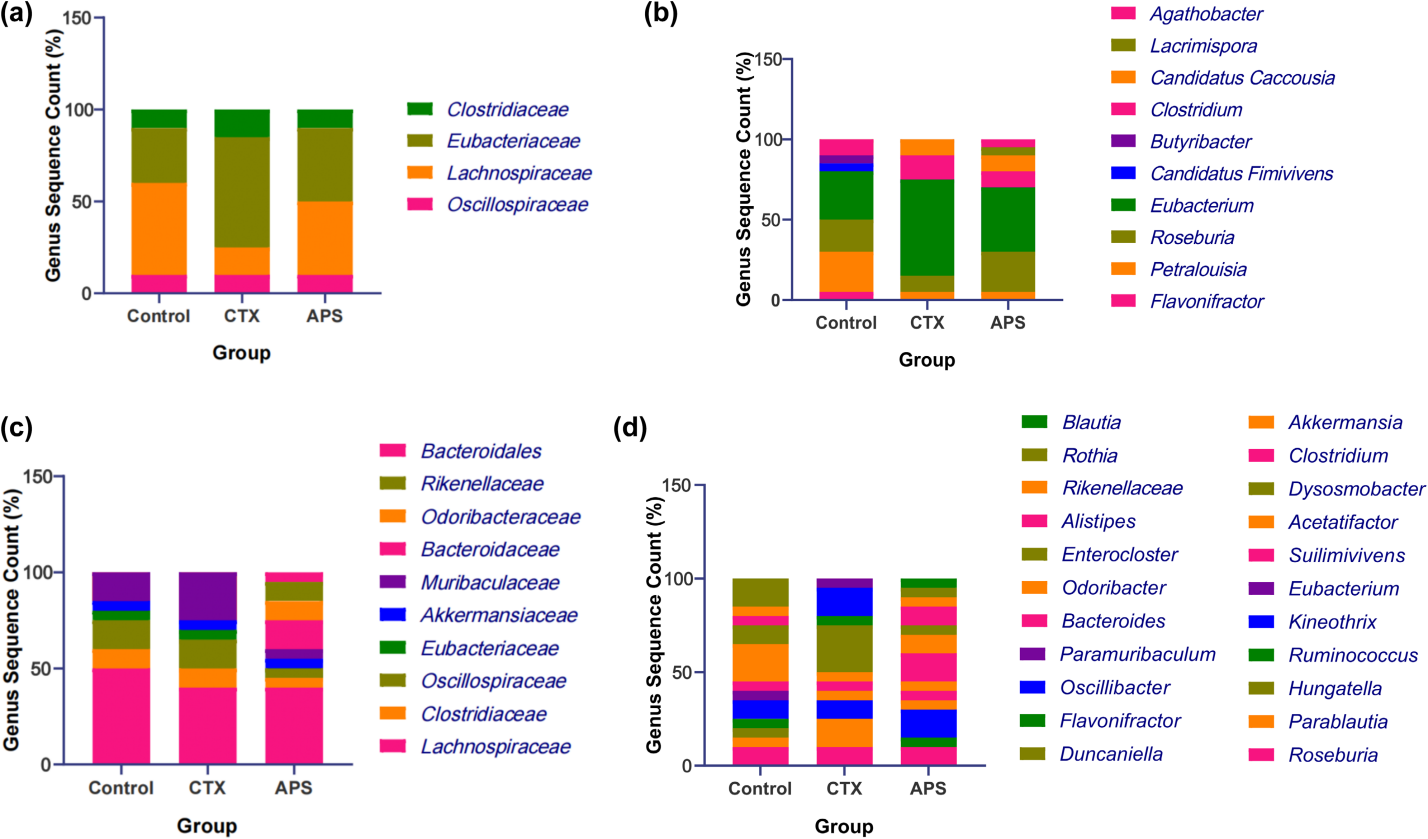
Changes in butyric acid‐producing functional microbial communities. (a, b) Statistical results at the genus level. The butyryl‐CoA: Acetate CoA‐transferase gene primers and the butyrate kinase primers, in that order. (c, d) Statistical results at the species level. The butyryl‐CoA: Acetate CoA‐transferase gene primers and the butyrate kinase primers, in that order.

Current research on polysaccharide‐butyrate gut microbiota tends to focus on the pharmacological actions of polysaccharides or butyrate, with mechanistic studies on polysaccharide‐microbiota‐butyrate interactions remaining at the macroscopic level (16S rRNA). Specific studies targeting mechanisms such as the enrichment of functional microbiota are still lacking. In this study, we obtained significantly different quantitative results for intestinal butyric acid levels, with the potential underlying principle being the corresponding changes in the functional bacteria responsible for butyrate production in the intestines. Therefore, we specifically enriched the genes encoding the two enzymes directly involved in butyrate production. The enrichment results of the butyryl‐CoA: acetate CoA‐transferase gene primers indicated a change in the structure of the butyrate‐producing gut microbiota in the mice at the genus level (Figure 5a) after CTX injection, showing a trend towards simplification, which was restored after oral APS administration. At the species level (Figure 5b), the number of butyrate‐producing bacterial species decreased from 7 to 5 after CTX injection but increased back to 7 after APS treatment. The enrichment results of the butyrate kinase primers at the genus level (Figure 5c) indicate a shift in the structure of the butyrate‐producing gut microbiota after CTX injection, with a decrease in the genus Lachnospiraceae and an increase in the genus Muribaculaceae. Following APS treatment, the number of butyrate‐producing bacterial genera in the intestine increased to nine, with four new butyrate‐producing bacterial genera not found in the control or CTX groups: Bacteroidales, Rikenellaceae, Odoribacteraceae, and Bacteroidaceae. At the species level (Figure 5d), the number of butyrate‐producing bacterial species in the intestines decreased from 12 to 10 after CTX injection, but increased to 13 after APS treatment, even exceeding the levels found in normal mice. In summary, oral administration of Astragalus polysaccharides reversed the damage to the butyrate‐producing functional gut microbiota caused by CTX and altered the structure, abundance, and diversity of the butyrate‐producing gut microbiota.

## 4 DISCUSSION

Recently, natural macromolecular polysaccharides have garnered widespread attention owing to their structural complexity and pharmacological activities (Zhang et al., 2022). It is noted that Chinese herbal medicine is one of the most significant sources of natural macromolecular polysaccharides (Yue et al., 2022). Astragalus polysaccharides are among the most representative tonifying polysaccharides in Chinese herbal medicines (Zhou et al., 2023). Whether the tonifying effects of astragalus polysaccharides are related to the metabolite butyric acid generated during its degradation by the intestinal microbiota has become a topic of interest in several studies (Liang et al., 2024; Wei et al., 2023). Therefore, we established a classic immunosuppression model for polysaccharide research using oral administration of astragalus polysaccharides as a background for immune activation in the body. We specifically investigated the changes in butyric acid levels in animal intestines at different stages, focusing on determining the underlying mechanism of these changes, namely, the variations in butyric acid‐producing functional microbial communities. The results indicated that oral treatment with astragalus polysaccharides led to a significant increase in intestinal butyric acid levels, primarily because of the adjustment effect of astragalus polysaccharides on the structure, abundance, and diversity of butyric acid‐producing functional microbial communities in the intestines. A key innovation of this study was the enrichment method employed for functional microbial communities in the intestine. We believe that this statistical method is more targeted and precise than the traditional 16S rRNA profiling (Zhang, Mao, et al., 2021). Consequently, our research confirms that the oral administration of astragalus polysaccharides induces an immune activation process accompanied by adjustments to butyric acid‐producing functional microbial communities in the intestines.

Presently, research on polysaccharide‐butyric acid interactions is mainly focused on the two areas themselves, leaving room for further exploration of the core factor of their correlation: microbial communities (Fu et al., 2019). The relationship between polysaccharides and microbial communities is initially manifested in certain Puls and gpPUL bacteria, which encode carbohydrate‐active enzyme genes that act on various polysaccharide substrates and break down complex macromolecular polysaccharides into monosaccharides and oligosaccharides. Subsequently, monosaccharides and oligosaccharides serve as substrates for the related functional microbial communities to break down and produce bioactive metabolites. Therefore, the diversity of functional microbial communities involved in the degradation of complex macromolecular polysaccharides is a key area worth exploring (Zhang, Wu, et al., 2023). Compared with the polysaccharide‐microbial community relationship, the association between microbial communities and butyric acid was clearer. Genes directly related to butyric acid production by butyrate‐producing bacteria include the butyryl‐CoA: acetate CoA transferase and butyryl‐CoA genes. In this study, we simultaneously used both gene primers to enrich microbial communities, which is a pioneering effort (Xiong et al., 2023). Our experimental results comprehensively demonstrated the changes in all butyric acid‐producing microbial communities in the intestines during the immune activation process induced by the oral administration of astragalus polysaccharides. Furthermore, studies have shown that butyric acid plays a significant role in maintaining intestinal barrier integrity, primarily by enhancing the tolerance of intestinal microbial communities and facilitating nutrient absorption, while also defending against pathogen invasion. This conclusion is supported by our research, indicating a potential link between the immune activation effects induced by astragalus polysaccharides through microbial degradation and the maintenance of intestinal barrier integrity.

An increasing number of clinical trials have systematically or conceptually supported the relevance of human intestinal microbiota to host health (Fan & Pedersen, 2021). Interventions in the intestinal microbiota to maintain intestinal homeostasis and influence disease treatment have become a key focus in the field of intestinal microbiota. This study provides a new prebiotic perspective. Astragalus polysaccharides, some of the most extensively studied traditional Chinese medicinal polysaccharides, have demonstrated pharmacological efficacy in animal experiments, livestock farming, and clinical trials (Li et al., 2023, Zhang, Zhang, et al., 2023). The greatest advantage of astragalus polysaccharides lies in their safety, which is primarily derived from plants with well‐defined components. Therefore, astragalus polysaccharides have the potential to serve as a prebiotic for targeted intervention in intestinal microbial communities (Huo et al., 2024). In this study, we presented the targeted intervention effect of astragalus polysaccharides on butyric acid‐producing microbial communities in the intestine, suggesting that further research could extend to other types of functional microbial communities in the intestine, such as propionic acid‐producing bacteria and lactic acid‐producing bacteria.

In conclusion, our study established and explored a polysaccharide‐microbial community‐butyric acid‐immune axis. We believe that any changes in any part of this biological axis could affect the overall system. Therefore, using polysaccharides to treat immune‐mediated diseases may be a promising approach.

## 5 CONCLUSION

In this study, we extracted and purified Astragalus containing the main chain of glucan. These homogeneous astragalus polysaccharides exhibited immunostimulatory effects in immunosuppressed mice induced by CTX injection, primarily manifested through the modulation of immune cell cytokines, immunoglobulins, and organ indices (thymus and spleen indices). During immunomodulation by astragalus polysaccharides, a significant difference in butyrate levels was observed in the mouse intestine. To explore this mechanism, we specifically enriched the functional bacteria responsible for butyrate production in the mouse intestine using two butyrate‐producing bacterial primers. These results showed that astragalus polysaccharides had a reparative effect on the disruption of the structure, abundance, and diversity of the butyrate‐producing gut microbiota caused by CTX injection, which may be one of the microbial mechanisms underlying the immunostimulatory effects of astragalus polysaccharides. Our study provides partial experimental evidence supporting the investigation of the potential prebiotic effects of astragalus polysaccharides and their further clinical applications. Further research into the potential prebiotic activity of astragalus polysaccharides, as well as the interaction mechanisms between astragalus polysaccharides and intestinal microbial communities, may prove to be a feasible strategy.

## AUTHOR CONTRIBUTIONS

**XinQian Rong:** Data curation (equal); formal analysis (equal); methodology (equal); project administration (equal); writing – original draft (equal); writing – review and editing (equal). **QingLong Shu:** Conceptualization (equal); funding acquisition (equal); methodology (equal); project administration (equal); software (equal); writing – review and editing (equal).

## CONFLICT OF INTEREST STATEMENT

The authors declare that they do not have any conflict of interest.

## ETHICS STATEMENT

This study was approved by the Experimental Animal Ethics Committee of Jiangxi University of Traditional Chinese Medicine (Ethics Committee No. JZLLSC20240306004).

## Data Availability

All data obtained or analyzed in this study can be obtained from the corresponding author.
